# Lunotriquetral Coalition: An Unusual Cause of Wrist Pain

**DOI:** 10.7759/cureus.5704

**Published:** 2019-09-19

**Authors:** Rabia Ali, Fahim Khan, Sikandar Saeed, Nismat Javed

**Affiliations:** 1 Miscellaneous, Shifa College of Medicine, Shifa Tameer-E-Millat University, Islamabad, PAK; 2 Orthopaedics and Traumatology, Shifa International Hospital, Islamabad, PAK; 3 Surgery, Shifa College of Medicine, Shifa Tameer-E-Millat University, Islamabad, PAK; 4 Internal Medicine, Shifa College of Medicine, Shifa Tameer-E-Millat University, Islamabad, PAK

**Keywords:** lunotriquetral coalition, congenital, carpal coalition, wrist pain

## Abstract

Carpal coalition is the fusion of two or more carpal bones in the wrist. This has a prevalence of 0.1% in Caucasian populations, with lunotriquetral coalition being the most common type. The incidence in Asian populations, including Pakistan, is not known. Usually, these fused carpal bones are asymptomatic. However, they can be the cause of undiagnosed wrist pain. We present the case of a 26-year-old female who presented with right-sided wrist pain. On X-ray, she was diagnosed to have a lunotriquetral coalition. We emphasize the role of radiographic imaging in cases of carpal coalition. Most patients are treated conservatively. However, in severe cases, surgical treatments may be considered.

## Introduction

According to the literature, the incidence of carpal coalitions in the general population is 0.1% with 90% of them being the fusion of lunate and triquetrum. The incidence of carpal coalitions is higher in African tribes [1]. Carpal coalitions follow an autosomal dominant route of inheritance [2,3]. Even though they are mostly asymptomatic, their non-osseous counterpart may show a range of symptoms, including - but not limited to - ulnar-sided wrist pain, ulnar nerve paresthesia, and an increased risk of degenerative disease [4].

De Villiers Minnaar developed a classification scheme for lunotriquetral coalition based on radiographic assessment, which is composed of four types. Minnaar’s classification focuses more on the osseous (types two and three) types of fusions, compiling all the non-osseous (type one) types into one category [5]. In this article, our aim is to present the case of a patient with the above condition, which, while not novel, warrants discussion due to its rarity in Pakistan.

## Case presentation

**Figure 1:**
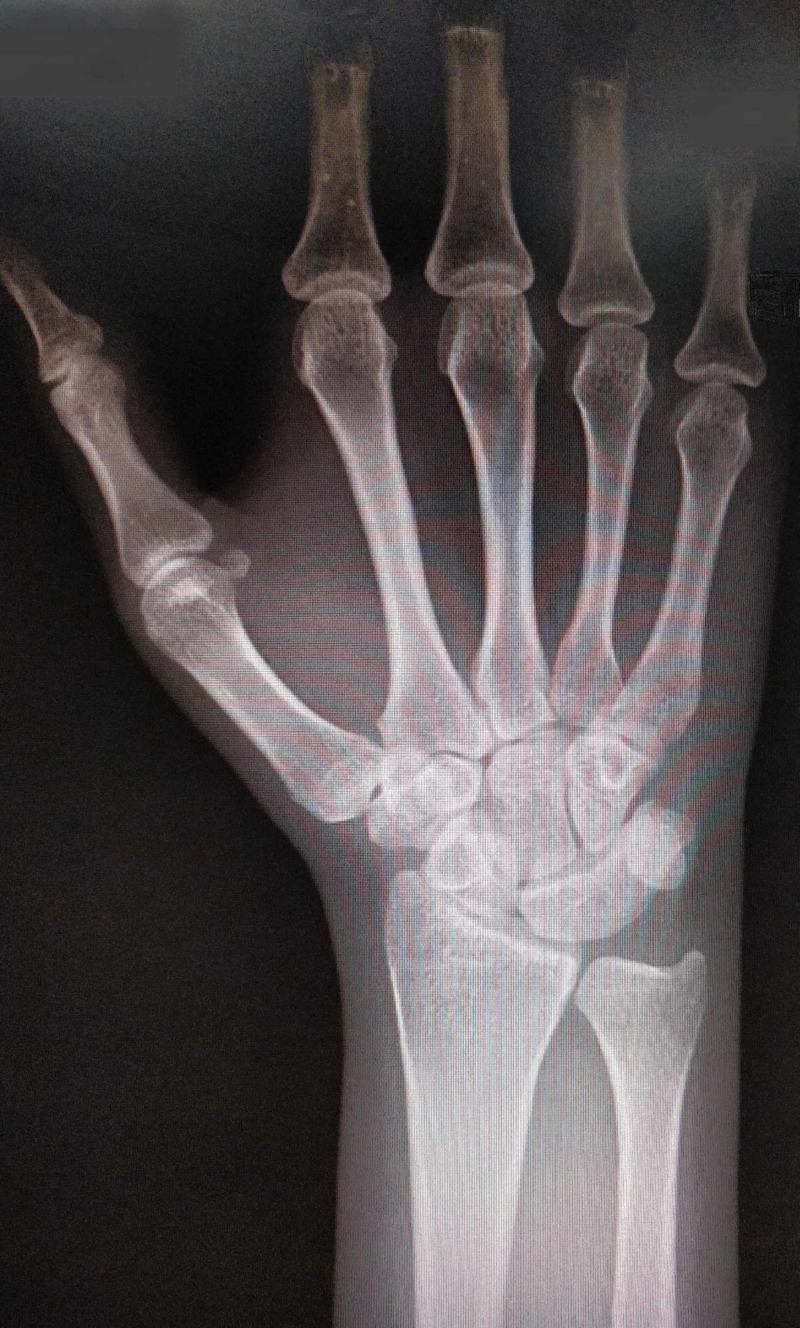
Anteroposterior radiograph

**Figure 2:**
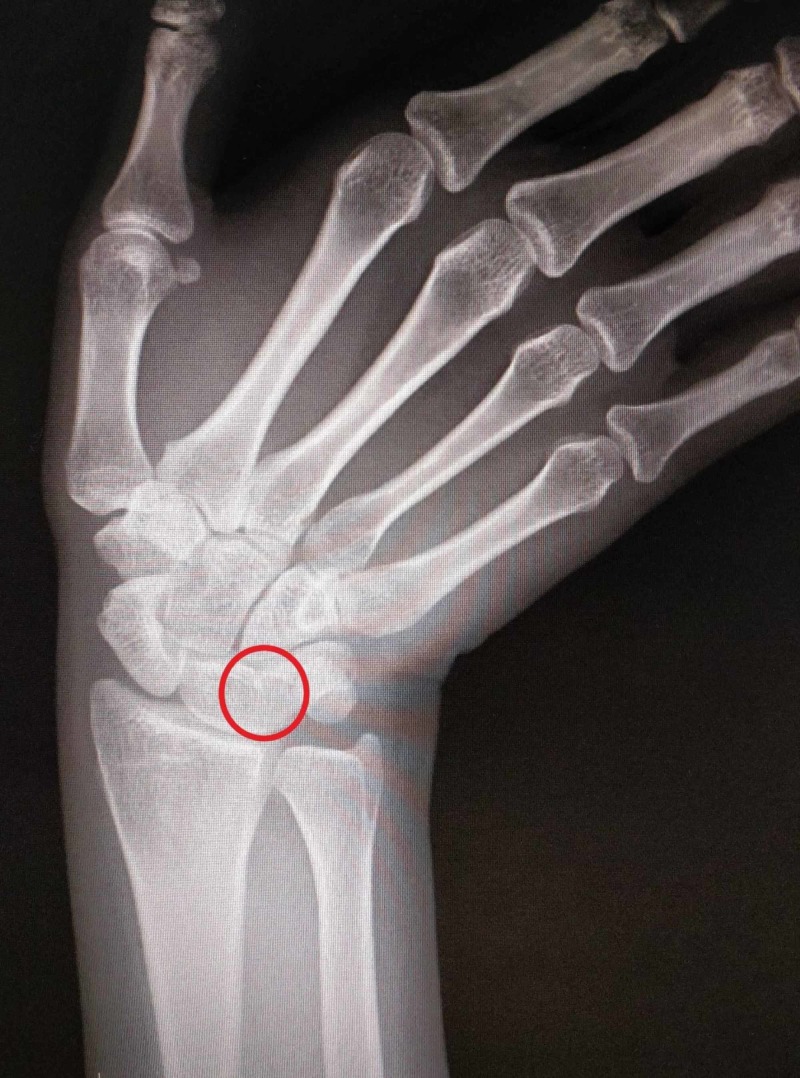
Lateral radiograph Coalition is marked in red circle

A 26-year-old woman presented to Shifa International Hospital Orthopedic outpatient clinic with complaints of right-sided wrist pain radiating towards the arm for one week, after being in a road traffic accident. The pain was reportedly mild to moderate in severity. On examination, mild diffuse tenderness was found over the ulnar aspect of the proximal carpal row. Ulnar deviation was reduced to 70% of normal, but normal ranges of flexion, extension and radial deviation were observed. The diagnosis was made with a hand grip dynamometer test which gave a reading of 20 kilograms on her affected hand and 30 kilograms on her left hand. On further investigations, a plain anteroposterior radiograph of the right wrist showed a right-sided osseous bridge between the lunate and triquetrum with a distal notch as shown in Figures 1, 2.

According to Minnaar’s classification, this is the characteristic of type two lunotriquetral coalition. She was prescribed oral analgesics and wrist immobilization in a splint for 10 to 14 days.

## Discussion

This case report highlights how patients who present with wrist pain should be assessed for lunotriquetral coalitions, provided that their history includes appropriate risk factors. Gender is a risk factor because this condition is more commonly found in females [6]. Ethnicity is another risk factor as African-Americans commonly present with this condition [6]. They arise embryologically due to the incomplete separation of cartilaginous zones of the neighboring carpals. Coalitions with a partial development of a central zone are considered to be incomplete fusions, whereas coalitions with no development of a central zone are complete fusions. One variant of the coalition is the presence of an osseous bridge as in our patient. Such a finding is associated with ulnar impaction syndrome that mimics the wrist pain that our patient presents with [7].

Carpal coalitions can be isolated incidences or associated with other congenital syndromes. Most isolated carpal coalitions are between the carpals of the same row [7]. Dyschondrosteosis, Banki syndrome and Turner syndrome are some of the few syndromes associated with lunotriquetral coalitions [8].

Anteroposterior and lateral X- ray images are usually sufficient to form a diagnosis [9]. Often there is expansion of the scaphoid-lunate joint space which we cannot see in our patient’s radiographs. A computed tomography (CT) can provide more information on the bone surfaces and is indicated for symptomatic patients [1,2]. However, Magnetic resonance imaging (MRI) is performed to have a clearer view of soft tissues such as the articular cartilage, and it gives a clearer image of pseudoarthrosis [9]. Our patient did not show any indication for a CT or an MRI to be done.

Lunotriquetral coalition is managed conservatively or surgically. Initially, immobilization, elevation and anti-inflammatory drugs are prescribed [2]. If surgical intervention is required, complete fusion of the coalition is accepted as one of the treatments. To that end, lunotriquetral coalition arthrodesis is performed [10]. However, carpal fusions can cause wrist instability and arthritis. Therefore, it is recommended to first exhaust all conservative managements before proceeding for surgical intervention. In some cases, wrist denervation can also be offered [2,10]. Our patient did not indicate any need for surgical intervention. She was asked to immobilize her wrist for a short period of time and to use appropriate analgesics. Her recovery was unremarkable.

## Conclusions

Wrist pain due to carpal coalitions is often misdiagnosed as wrist sprain, and its true incidence is yet to be determined because they are often asymptomatic. Moreover, the data on Asian populations - especially Pakistan - are scarce, so incidence in the region is completely unknown. However, the fact that they can be a cause of discomfort means that orthopedics should consider this condition in their differential diagnoses when a patient presents with unexplained wrist pain.
